# Frequency of non-alcoholic fatty liver disease (NAFLD) and its associated risk factors among Type-2 diabetics

**DOI:** 10.12669/pjms.38.1.4968

**Published:** 2022

**Authors:** Alia Ali, Muhammad Joher Amin, Muhammad Uthman Ahmed, Azeem Taj, Muhammad Aasim, Elsa Tabrez

**Affiliations:** 1 Dr. Alia Ali, FCPS. Assistant Professor, Department of Medicine, Shaikh Zayed Postgraduate Medical Institute, Lahore, Pakistan; 2 Prof. Muhammad Joher Amin, FCPS. Department of Gastroenterology, Shaikh Zayed Postgraduate Medical Institute, Lahore, Pakistan; 3 Prof. Muhammad Uthman Ahmed, FCPS. Department of Medicine, Shaikh Zayed Postgraduate Medical Institute, Lahore, Pakistan; 4 Prof. Azeem Taj, FCPS. Department of Medicine, Shaikh Zayed Postgraduate Medical Institute, Lahore, Pakistan; 5 Mr. Muhammad Aasim, Statistician, Shaikh Zayed Postgraduate Medical Institute, Lahore, Pakistan; 6 Elsa Tabrez, Undergraduate Student, New York Medical College, New York, USA

**Keywords:** NAFLD, BMI, Type-2 Diabetes

## Abstract

**Objectives:**

To determine the frequency of Non-Alcoholic Fatty Liver Disease (NAFLD) and its associated risk factors among Type-2 Diabetic patients.

**Methods:**

This cross-sectional study was conducted in Diabetic Clinic of Shaikh Zayed Postgraduate Medical Institute Lahore from September 2019-February 2020. Type-2 diabetics regardless of age were divided into two groups, one with fatty liver disease and the other without this, evaluated by Abdominal Ultrasonography and were further evaluated by measurement of BMI, obesity, HbA1c and lipid profile. Exclusion criteria were patients having history of or currently taking alcohol, chronic Liver Disease of any cause and intake of hepatotoxic drugs. Qualitative measures were compared between groups by using Chi-square test. Binary logistic regression was used to see the association of factors with fatty liver disease. P-value ≤ 0.05 was considered significant.

**Results:**

A total of 185 subjects were included in the study with the mean age of 53.0±9.0 years. About 54.6% patients were diagnosed to have fatty liver disease. When compared the cases with and without fatty liver disease, age and HDL cholesterol had no significant difference between groups while other measures like BMI, TGs & cholesterol levels, ALT and AST were significantly higher among cases with NAFLD. BMI >24.5, HbA1c >7.0 and ALT >40.0 can predict NAFLD among Type-2 diabetic patients with 96.8% accuracy.

**Conclusion:**

There is high prevalence of NAFLD among Type-2 diabetic patients and strong association between Type-2 diabetics with NAFLD and risk factors like; obesity, high HbA1c, hyperlipidemia and high ALT. Therefore, early recognition by ultrasonography in high risk patients and intervention like life style modification, maintenance of healthy weight, obesity prevention, treatment of dyslipidemia and good glycemic control should be achieved in such subjects and can prevent NAFLD.

## INTRODUCTION

Non-Alcoholic Fatty Liver Disease (NAFLD) is the most common chronic liver disease.^1^ It is a condition of hepatic steatosis without significant alcohol intake or another identifiable secondary cause that would result in fat accumulation within hepatocytes. NAFLD is recognized as an important public health problem nowadays. NAFLD encompasses a variety of liver pathologies including simple steatosis, Non-Alcoholic Steato-Hepatitis (NASH), fibrosis, cirrhosis and finally cancer.^2^

It was once believed to be a benign condition that only rarely progressed to chronic liver disease; steato-hepatitis may progress to liver fibrosis and cirrhosis and may result in liver related morbidity and mortality.^3^

Global prevalence of NAFLD is 25.24% with highest prevalence in Middle East and South America and lowest in Africa.^4^ The overall prevalence of NAFLD in western countries varies from 15-40% and in Asian countries from 9-40%.^5^ The overall prevalence of NAFLD in Asia is now estimated to be 29.6% and may have surpassed that in Western populations.^6^

The principal causes of NAFLD are obesity (present in 40% or more of affected patients), diabetes mellitus (in 20% or more), and hypertriglyceridemia (in 20% or more) in association with insulin resistance as part of the metabolic syndrome.^7^ The increasing prevalence of NAFLD mirrors that of obesity and Type-2 diabetes over the last two decades.^8^ The growing burden of NAFLD parallels the increasing prevalence of obesity in Asia. Overweight/obesity and insulin resistance have been strongly linked with NAFLD.^9^ The prevalence of Type-2 diabetes in Pakistan is 17.1% while prevalence of NAFLD in Pakistan and prevalence of NAFLD in type2 diabetics in Pakistan are 14% and 32-72% respectively.^10^

NAFLD is now recognized as the most prevalent chronic liver disease worldwide. Type-2 diabetes is an important risk factor for NAFLD.^11^ Its prevalence worldwide is thought to be approximately 20% in the general population and up to 70% in patients with Type-2 diabetes mellitus.^12^,^13^ and 20-30% have the more severe form of the disease with lobular inflammation and hepatocyte ballooning (nonalcoholic steatohepatitis, NASH).^14^

The presence of both NAFLD and Type-2 DM increases the likelihood of the development of complications of diabetes (including both macro and micro vascular complications) as well as augmenting the risk of more severe NAFLD, including cirrhosis, hepatocellular carcinoma and death.^14^ Although long term prospective studies are lacking, these patients are also believed to be at higher risk of disease progression to advanced fibrosis and cirrhosis.^14^

Despite the high prevalence and serious clinical implications, NAFLD is usually overlooked in clinical practice. The aim of the current study was to determine the prevalence of NAFLD in a large, randomly selected population of people with Type-2 DM, using Ultrasound grading classification to confirm fatty liver and to examine correlation of NAFLD with other risk factors like obesity and hyperlipidemia in such subjects.

## METHODS

A total of 185 Type-2 diabetics with age between 25 and 84 with 133(61.1%) males, who attended the diabetic outdoor clinic of Shaikh Zayed Hospital, Lahore Pakistan from September 2019-February 2020 for routine follow-ups were included in the study. Informed consent was obtained from each patient. A standard questionnaire regarding the demographic data such as age, gender, height, body weight; while wearing lightweight clothing, without shoes, and family history of diabetes mellitus. Blood pressure, smoking habits were recorded for each patient. BMI was calculated as weight (Kg) divided by height (m^2^). The normal range is 18.5-22.9 Kg/m^2^. Adults are overweight if BMI is ≥ 25Kg/m^2^, pre-obese if 23-24.9 Kg/m^2^ and obese if it ≥ 30Kg/m^2^.

Metabolic parameters such as glycosylated hemoglobin (HbA1c), ALT, AST and lipid profile (total cholesterol, triglycerides, LDL and HDL) were done. Patients with one of the criteria: LDL-C cholesterol>100mg/dl, Total cholesterol >200mg/dl, triglycerides > 150mg/dl and HDL-C <40 mg/dl in males and <50mg/dl in females were considered to have dyslipidemia.

According to American Diabetic Association, HbA1c<5.7% normal, 5.7% and <6.5% prediabetes and 6.5% or higher diabetes range. The levels of AST and ALT were evaluated by collection of venous blood samples and serum biochemistry performed at one designated laboratory. ALT<63 and AST <37 were taken as normal. The patients with criteria such as history of alcohol consumption, chronic liver disease of any other cause, intake of hepatotoxic drugs and pregnant, lactating mothers were excluded.

The diagnosis of NAFLD currently requires: (1) evidence of hepatic steatosis (HS) by imaging or histology, (2) no alcohol consumption, (3) no competing cause of HS, and (4) no coexisting causes of chronic liver disease.^15^

To detect fatty changes in liver, all patients were enrolled and underwent ultrasonography, performed by single experienced radiologist (to prevent interpersonal variation), using a high-resolution B-mode ultrasonography system having an electric convex transducer mid frequency of 5-7 MHZ.

NAFLD was classified based on the standard ultrasonographic criteria. This classification was adopted and already tested by other investigators.^15^ Grade 0, no steatosis (liver and renal cortex of the same echogenicity); Grade-1, mild steatosis(slightly brighter liver as compared to the renal cortex, clear visualization of diaphragm, and interface of hepatic veins with sharp contours); Grade-2, moderate steatosis(brighter liver with attenuated US beam at deeper parts of the liver, diaphragm, and hepatic veins still visible but with blunted contours); Grade 3, severe steatosis(very bright liver, severe US beam attenuation, diaphragm, or hepatic veins not visible).

It was a cross-sectional analytical study. Sample size of 185 was estimated by 95% confidence level and 7% margin of error with expected prevalence of fatty liver as 64.0%.^17^ Data were managed through SPSS 20.0. For quantitative measures median (IQR) were used except for age where mean±SD was used to describe the data overall as well as in groups as except age all data were skewed. Independent sample t-test was used to compare age between two groups and one way ANOVA to compare among three groups. Mann Whitney U test was used for all other quantitative measures to compare between two groups and Kruskal Wallis test among three groups. Receiver Operative Characteristics (ROC) curve was used to find cut-offs for various quantitative measures. Qualitative measures were compared between groups by using chi-square test. Binary logistic regression was used to see the association of factors with fatty liver disease and results were presented by using adjusted odds ratios with 95% confidence interval. P-value ≤0.05 was considered significant.

### Ethical approval:

(Ref: F.39/NHRC/Admin/IRB/53, Dated: 13-02-2019).

## RESULTS

The Study was conducted on 185 diabetic patients with an average age of 53.0±9.0 years. Among them 113(61.1%) were males, 148(80.0%) had positive family history of diabetes, 56(30.3%) were smokers, 76(41.1%) had hypertension also and 101(54.6%) were diagnosed to have fatty liver disease. The median BMI was 27.0(23.0 – 30.0) and HbA1c was 7.0(6.7 – 8.0). Total cholesterol, triglycerides, LDL cholesterol and HDL cholesterol were measured to be 210(185 – 249), 180(150 – 200), 110(100 – 144) and 43(40 – 46) mg/dl respectively. The median ALT and AST levels were 39(35 – 78) and 33(29 – 37) respectively. Among fatty liver disease cases, 69(68.3%) were of grade-1, 29(28.7%) of grade-2 and 3(3.0%) of grade-3.

When compared the cases with and without fatty liver disease, age and HDL cholesterol had no significant difference between groups with p-values 0.971 and 0.319 respectively while all other measures like BMI, other cholesterol levels, ALT and AST were significantly higher among cases with NAFLD. The gender, family history, smoking and hypertension had no significant difference between two groups (Table-I).

**Table 1:** Comparison of characteristics between groups with and without NAFLD.

	NAFLD(n=101)	Normal(n=84)	P-value
Age (years)	53 ± 9.0	53 ± 10	0.971
BMI (kg/m^2^)	29.0 (27.0 – 31.0)	23.0(22.0 – 24.0)	<0.001
HbA1c (%)	8.0 (7.5 – 8.9)	6.7(6.5 – 6.8)	<0.001
Cholesterol (mg/dl)	230(207 – 276)	187(160 – 214)	<0.001
TGs (mg/dl)	195(170 – 214)	155(130 – 183)	<0.001
LDL Cholesterol (mg/dl)	120(100 – 155)	100(95 – 120)	<0.001
HDL Cholesterol (mg/dl)	43(41 – 46)	43(40 – 47)	0.319
ALT (IU/L)	70(40 – 90)	35(32 – 38)	<0.001
AST (IU/L)	35(31 – 39)	30(28 – 33)	<0.001
Male	57(56.4)	56(66.7)	0.204
F/H DM	82(81.2)	66(78.6)	0.796
Smoker	36(35.6)	20(23.8)	0.113
HTN	41(40.6)	35(41.7)	1.000

**Test applied for comparison:** T-Test for values expressed as Mean ± SD, Mann Whitney U test for values expressed as median (Q_1_ – Q_3_), Chi-square test for values expressed as n(%).

**Fig.1:**
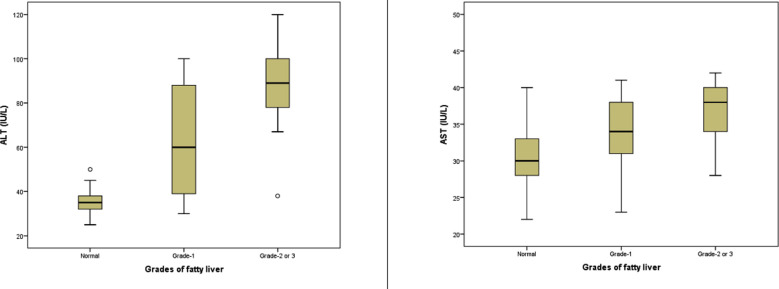
The distribution of ALT and AST among three groups by NAFLD category.

Further investigation was made if the grades of disease had any difference as compared to normal and each other. For this purpose, the three cases of grade-3 were merged with grade-2 cases. It was observed again the age and HDL had no difference between grades, family history, smoking and hypertension also had no significant difference but gender showed a significant difference with high percent of males among grade-2/3 cases and low percentage in grade-1 cases with a p-value 0.013. The ALT and AST were the only variables that showed a significant difference between grade-1 and grade-2/3 cases, while other variables had no difference between grade-1 and grade-2/3, though both groups had significantly higher averages as compared to normal group. The family history of diabetes, smoking status and hypertension status also had no significant difference among three groups. (Table-II, Fig.1)

**Table II:** Comparison of characteristics among groups by grades of NAFLD.

	Normal	NAFLD grade-1	Grade- 2/3	P-value
Age	53±10	53±9	54±8	0.696
BMI	23(22–24)^a^	29(27–32)^b^	29(27–31)^bc^	<0.001
HbA1c	6.7(6.5–6.8) ^a^	8(7.5–9) ^b^	7.95(7.1–8.5)^bc^	<0.001
Cholesterol	189(160–217) ^a^	230(207–272) ^b^	230(203.5–280)^bc^	<0.001
TGs	155(130–185) ^a^	195(180–220) ^b^	187.5(157.5–200)^bc^	<0.001
LDL Cholesterol	100(95–120) ^a^	120(100–155) ^b^	116(100–157.5)^bc^	0.002
HDL Cholesterol	42.5(40–46) ^a^	42(40–46) ^ab^	43(41–48.5)^abc^	0.462
ALT	35(32–38) ^a^	60(39–88) ^b^	89(78–100) ^c^	<0.001
AST	30(28–33) ^a^	34(31–38) ^b^	38(34–40) ^c^	<0.001
Male	57(66.3)	32(47.8)	24(75.0)	0.013
H/O DM	68(79.1)	54(80.6)	26(81.2)	0.955
Smoker	21(24.4)	22(32.8)	13(40.6)	0.202
HTN	35(40.7)	33(49.3)	8(25.0)	0.065

Test applied for comparison among three groups is one way ANOVA for values expressed as MEAN ± SD

Kruskal Wallis and Mann Whitney U test for values expressed as median (Q_1_ – Q_3_)

Chi-square test for values expressed as n (%)

The difference between group averages with uncommon letter in superscript are significant.

**Fig.2:**
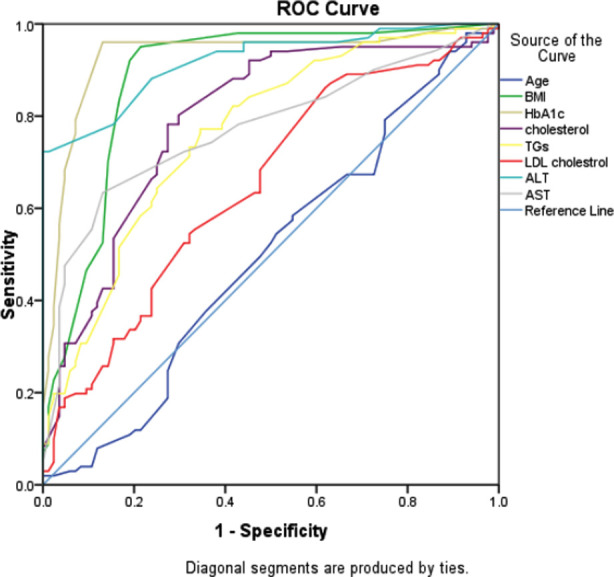
ROC curve defining area under the curve for different measures to identify the NAFLD.

By ROC curve BMI>24.5, HbA1c >7.0 and ALT>40.0 BMI, HbA1c and ALT were the three variables identified through ROC curve with respective cutoffs of > 24.5, >7.0 and >40.0 to predict NAFLD most accurately. When binary logistic regression model was fitted along other variables with backward Wald method, these three predictors were significant and gender, smoking, hypertension and family history were insignificant (so eliminated at 5^th^ step model). The adjusted odds ratio of BMI>24.5 and HbA1c>7.0 were almost around 31.0 while the adjusted odds ratio for ALT was 332.76(22.1 – 5017.9) and 98.0% of the fatty liver cases and 95.2% of non-fatty liver cases were identified accurately by these three measures with cutoffs given (Table-III, Fig.2).

**Table III:** Binary logistic regression model with predictive accuracies and adjusted odds ratios along 95% confidence interval).

	P-value	Adjusted Odds ratio (95% CI)	Predictive accuracy of binary logistic regression model				
	
					Predicted		
	
BMI> 24.5	0.003	30.9 (3.3 – 293.1)			Fatty Liver	Normal	Correct%
HbA1c > 7.0	< 0.001	30.9 (6.5 – 147.5)	Observed	Fatty Liver	99	2	98.0
ALT > 40.0	< 0.001	332.8 (22.1 – 5017.9)		Normal	4	80	95.2
Constant	< 0.001	0.000	Overall percentage				96.8

a. Variable(s) entered on step 1: Gender, F/H DM, Smoker, HTN, BMI>24.5, HbA1c> 7.0, ALT>40.0.

The BMI>24.5, HbA1c>7.0 and ALT > 40.0 can predict NAFLD among diabetic patients with 96.8% accuracy.

## DISCUSSION

In our study we observed high prevalence of fatty liver disease among Type-2 diabetic patients. Out of 185 Type-2 diabetic patients 101(54.6%) were diagnosed to have fatty liver disease and 69(68.3%) out of these were of Grade-1, 29 (28.7%) of Grade-2 and 3(3.0%) of Grade-3 fatty liver disease. A study conducted by Rao et al. also showed high prevalence (64.2%) of NAFLD among Type-2 diabetic patients and also among these, more patients had Grade-1 fatty liver.^18^ Similarly Patel et al. also showed higher frequency of NAFLD among Type-2 diabetic female patients as compared to male.^17^ Solmalwar AM et al. also showed similar results with higher frequency of NAFLD (56.66%)among Type-2 diabetic patients also showing increased frequency of microvascular complications (retinopathy 67.67%, neuropathy 52.94%, nephropathy 83.82%) among these patients.^19^

Also in Edinburgh Type-2 diabetic study, Rachel M et al. showed higher frequency of NAFLD (56.9%) among Type-2 diabetics.^20^ Our study also showed that BMI, HbA1c and ALT could predict NAFLD among diabetic patients with 96.8% accuracy. Similarly, Somalwar et al. showed BMI, HbA1c(7.86) and elevated liver enzymes to be significantly associated with Type-2 diabetics having fatty liver as compared to those with normal liver.^19^ A meta-analysis by Nasrin et al. showed high BMI (29%)in Type-2 diabetic patients with NAFLD.^21^ Similarly Bhatt K et al. found that BMI was significantly higher in patients with NAFLD(28.27%) than a control group (26.19%) without fatty liver.^22^ Aqeela et al. showed NAFLD is more prevalent in patients with uncontrolled Type-2 diabetes (high HbA1c levels) 98% as compared to controlled disease 2%.^23^

Our study also showed raised ALT levels in patients with NAFLD as compared to those without NAFLD. Lu et al. reported the prevalence of NAFLD in Type-2 diabetics was significantly associated with elevated ALT levels.^24^ Similarly, Prabharkar A et al. showed that elevated liver enzymes, HbA1c and obesity were all significantly associated with NAFLD.^25^

Contrary to our study Ijaz et al. showed in their study that raised ALT and AST were not a common finding in Type-2 diabetics having NAFLD.^26^ Similarly Gabriele et al. showed in their study that ALT levels are not predictable of NAFLD in Type-2 diabetics.^27^ The prevalence of NAFLD in Type-2 diabetes mellitus continues to be overlooked by clinicians, but there is an increased awareness about the negative health consequences of steatohepatitis. As seen from multiple studies mentioned above obese Type-2 diabetics are more prone to develop NAFLD, this issue highlights the importance of including weight management in any strategy targeting the epidemic of NAFLD.

Early recognition and intervention are key to improving clinical outcomes and reducing the economic and health care burden of NAFLD.^15^ Lifestyle intervention with diet low in carbohydrates especially sugars and refined carbohydrates and increased monounsaturated and omega 3 fatty acid intake, exercise like aerobic, resistance or high intensity intermittent, appear to have beneficial effects. Behavioral modification in the form of eating less, more physical activity and avoiding sedentary lifestyle is the initial step in managing Type-2 diabetes, this also applies to patients with NAFLD.^28^,^29^

### Limitations of the study:

Due to resources constraint the sample size was calculated with 7% margin of error. A study with larger sample size is advised.

## CONCLUSION

There is high prevalence of NAFLD among Type-2 diabetic patients and strong association between Type-2 diabetics with NAFLD and risk factors like; obesity, high HbA1c, hyperlipidemia and high ALT. Therefore, early recognition by noninvasive tool ultrasonography, especially in high-risk patients and intervention like life style modification of such subjects can prevent occurrence of NAFLD in future.

Moreover, strong association between Type-2 diabetics with NAFLD and obesity, dyslipidemia and uncontrolled diabetes suggest that our priority in such patients should be obesity prevention with healthy diet and exercise, treatment of dyslipidemia and good glycemic control respectively.

### Authors Contributions:

**AA, MJA, MUA:** Conceived, designed, data collection and did editing manuscript.

**MA:** Did statistical analysis.

**AA, ET:** Did manuscript writing. AT: did review and final approval of manuscript.

**AA**: Responsible and accountable for accuracy of the work.
